# POU6F1 Expression Predicts Favorable Prognosis in Lung Adenocarcinoma: Validation Using Patient Cohort and TCGA Data

**DOI:** 10.3390/cimb48050456

**Published:** 2026-04-28

**Authors:** Mincheol Chae, Jae-Ho Lee, Hyowon Hong, Dong Yoon Keum, Deok Heon Lee

**Affiliations:** 1 Department of Thoracic and Cardiovascular Surgery, Keimyung University Dongsan Hospital, Keimyung University School of Medicine, Daegu 42403, Republic of Korea; ysacmc@dsmc.or.kr (M.C.); kdy@dsmc.or.kr (D.Y.K.); 2 Department of Anatomy, Keimyung University School of Medicine, Daegu 42403, Republic of Korea; anato82@dsmc.or.kr (J.-H.L.);; 3 Department of Thoracic and Cardiovascular Surgery, School of Medicine, Kyungpook National University Hospital, Kyungpook National University, 130 Dongdeok-ro, Jung-gu, Daegu 41944, Republic of Korea

**Keywords:** POU6F1, non-small cell lung cancer, lung adenocarcinoma, lung squamous cell carcinoma

## Abstract

Non-small cell lung cancer (NSCLC) is the most common type of lung cancer and remains a leading cause of cancer-related mortality worldwide. Lung adenocarcinoma (AD) and lung squamous cell carcinoma (SCC) represent the two major histological subtypes with distinct molecular characteristics. POU domain class 6 transcription factor 1 (POU6F1) is involved in gene regulation and cellular differentiation, but its clinical significance in NSCLC remains unclear. This study investigated the clinicopathologic and prognostic significance of POU6F1 expression in NSCLC. POU6F1 mRNA expression was analyzed in tumor tissues obtained from 153 patients with NSCLC using quantitative real-time polymerase chain reaction. The associations between POU6F1 expression and clinicopathological characteristics were evaluated, and survival analysis was performed to determine its prognostic value. In addition, publicly available datasets from The Cancer Genome Atlas (TCGA) were analyzed to validate the clinical significance of POU6F1 expression. High POU6F1 expression was observed in 48 patients (31.4%), whereas 105 patients (68.6%) showed low expression. High POU6F1 expression was significantly associated with younger age (*p* = 0.027), female sex (*p* = 0.041), non-smoking status (*p* = 0.002), adenocarcinoma histology (*p* = 0.021), and the presence of EGFR mutations (*p* = 0.038). Survival analysis demonstrated that high POU6F1 expression was associated with improved overall survival in patients with NSCLC (*p* = 0.015). When stratified by histological subtype, higher POU6F1 expression was associated with better survival outcomes in AD but not in SCC. Analysis of TCGA datasets confirmed that elevated POU6F1 expression was significantly associated with favorable survival in AD, whereas no significant prognostic value was observed in SCC. POU6F1 expression is significantly associated with clinicopathological characteristics and improved survival in patients with lung AD. Multivariate Cox regression analysis further confirmed that POU6F1 expression was an independent prognostic factor for overall survival. These findings suggest that POU6F1 may serve as a potential prognostic biomarker based on mRNA expression in NSCLC, particularly in AD. Further studies are warranted to validate these findings at the protein level and to elucidate the underlying biological mechanisms.

## 1. Introduction

Lung cancer remains the leading cause of cancer-related mortality worldwide and represents a major global health burden [1,2]. Among lung cancers, non-small cell lung cancer (NSCLC) accounts for approximately 85% of all cases and is mainly classified into two major histological subtypes: lung adenocarcinoma (AD) and lung squamous cell carcinoma (SCC) [3]. Despite substantial advances in surgical techniques, targeted therapies, and immunotherapy, the overall prognosis of patients with NSCLC remains poor [4,5]. This is largely attributable to late diagnosis, tumor heterogeneity, and the development of therapeutic resistance. Therefore, the identification of reliable molecular biomarkers associated with tumor progression and patient prognosis is essential for improving clinical management and developing novel therapeutic strategies.

Recent studies have emphasized the critical role of transcription factors in cancer development and progression [6,7]. Dysregulation of transcriptional control is widely recognized as a hallmark of tumorigenesis and can influence various cellular processes, including cell proliferation, apoptosis, invasion, and metastasis. Members of the POU (Pit-Oct-Unc) family of transcription factors play essential roles in cellular differentiation, development, and gene regulation [8]. Among these factors, POU domain class 6 transcription factor 1 (POU6F1), also known as BRN5 or TCFB1, exhibits tissue-specific expression and has been primarily implicated in neuronal development and differentiation [9,10,11]. Emerging evidence suggests that POU6F1 may also contribute to tumorigenesis. Previous studies have reported that altered expression of POU6F1 is associated with cancer progression and clinical outcomes in several malignancies [10,11]. In lung adenocarcinoma, reduced POU6F1 expression has been shown to suppress tumor proliferation by regulating the HIF1A signaling pathway and has been associated with unfavorable patient prognosis [12]. However, the clinical significance of POU6F1 expression in NSCLC, particularly with respect to differences between adenocarcinoma and squamous cell carcinoma, remains largely unclear.

The Cancer Genome Atlas (TCGA) provides comprehensive genomic and transcriptomic datasets that have greatly facilitated the identification of potential biomarkers in various cancers. Analyses of TCGA datasets allow researchers to investigate gene expression patterns and their associations with clinical outcomes [13]. Our preliminary analysis of TCGA data suggested that POU6F1 expression may have prognostic relevance in NSCLC.

Therefore, the present study aimed to investigate the clinical and prognostic significance of POU6F1 gene expression in NSCLC. We analyzed POU6F1 mRNA expression levels in tumor tissues obtained from Korean patients with NSCLC using quantitative real-time polymerase chain reaction (qRT-PCR). We further evaluated the associations between POU6F1 expression and clinicopathological characteristics and performed survival analyses to determine its prognostic value. Additionally, TCGA datasets were analyzed to validate the clinical significance of POU6F1 expression. Our findings may provide new insights into the potential role of POU6F1 as a prognostic biomarker in NSCLC.

## 2. Materials and Methods

### 2.1. Patients and Tissue Samples

A total of 153 patients (mean age, 67.27 ± 8.12 years; range, 42–86 years) diagnosed with non-small cell lung cancer (NSCLC) were included in this study. Paired tumor tissues and adjacent non-tumorous lung tissues were obtained from the Keimyung Human Bioresource Bank (Daegu, Republic of Korea). All patients underwent surgical resection at Keimyung University Dongsan Medical Center (Daegu, Republic of Korea) between April 2010 and January 2016. Clinicopathological data were retrospectively reviewed from medical records. Tumor staging was determined according to the 8th edition of the American Joint Committee on Cancer (AJCC) TNM staging system. This study was conducted in accordance with the principles of the Declaration of Helsinki. Paired tumor tissues and adjacent non-tumorous lung tissues were obtained from the Keimyung Human BioResource Bank (Daegu, Republic of Korea). Clinical data were retrospectively reviewed from medical records. The study used de-identified human specimens and clinical data provided by the biobank. The study protocol was approved by the Institutional Review Board of Keimyung University Dongsan Medical Center (IRB No. 2020-07-027).

### 2.2. RNA Isolation and mRNA Expression Analysis

Total RNA was extracted from tissue samples using TRIzol reagent (Molecular Research Center Inc., Cincinnati, OH, USA) according to the manufacturer’s instructions. RNA concentration and purity were measured using a NanoDrop 1000 spectrophotometer (Thermo Scientific, Wilmington, DE, USA).

Complementary DNA (cDNA) was synthesized from 2 μg of total RNA using Moloney murine leukemia virus (M-MLV) reverse transcriptase (Promega, Madison, WI, USA). Quantitative real-time polymerase chain reaction (qRT-PCR) was performed using gene-specific primers as previously described [12].

The relative mRNA expression level of POU6F1 was normalized to β-actin as an internal control and calculated using the 2^−ΔΔCt^ method. Each sample was analyzed in triplicate, and five serially diluted control samples were included in each experiment to ensure assay reliability.

### 2.3. TCGA Data Analysis

Gene expression and clinical data for NSCLC were obtained from The Cancer Genome Atlas (TCGA) data portal in March 2021. The TCGA dataset included a total of 1130 samples, consisting of 1019 primary tumor tissues (517 adenocarcinoma and 502 squamous cell carcinoma) and 111 adjacent normal tissues. For RNA sequencing data analysis, datasets corresponding to adenocarcinoma (AD) and squamous cell carcinoma (SCC) were extracted separately. POU6F1 mRNA expression levels were evaluated together with relevant clinical parameters. Overall survival (OS) was defined as the time from the date of surgery to death from any cause or the last follow-up. This study complied with the TCGA publication guidelines for the use of publicly available datasets.

### 2.4. Statistical Analysis

All statistical analyses were performed using the Statistical Package for the Social Sciences (SPSS) software (version 24.0; IBM Corp., Armonk, NY, USA). Associations between clinicopathological variables were analyzed using the chi-square test or the Mann–Whitney U test. For survival analysis, the mean expression level of POU6F1 was used as the cutoff value to classify patients into high-expression and low-expression groups. Survival curves were generated using the Kaplan–Meier method, and differences between groups were assessed using the log-rank test. Variables that were statistically significant in univariate analysis were included in the multivariate Cox regression model. Hazard ratios (HRs) and 95% confidence intervals (CIs) were calculated. Multivariate Cox regression analysis was performed to evaluate independent prognostic factors for overall survival. A two-tailed *p* value of <0.05 was considered statistically significant.

## 3. Results

### 3.1. POU6F1 Expression in NSCLC Patients

POU6F1 expression was successfully analyzed in 153 patients, with a mean expression value of 0.81 ± 0.53. The clinicopathological characteristics according to POU6F1 mRNA expression are summarized in Table 1. Among the total cohort, high POU6F1 expression was observed in 48 patients (31.4%), whereas 105 patients (68.6%) showed low expression. Statistical analysis revealed that high POU6F1 expression was significantly associated with younger age (*p* = 0.027), female sex (*p* = 0.041), and a non-smoking history (*p* = 0.002). Furthermore, POU6F1 expression differed significantly according to histological subtype (*p* = 0.021), with higher expression observed in adenocarcinoma compared with squamous cell carcinoma. A significant association was also identified between high POU6F1 expression and the presence of EGFR mutations (*p* = 0.038). In contrast, no significant associations were observed between POU6F1 expression and other tumor characteristics, including T stage (*p* = 0.351), N stage (*p* = 0.449), overall pathological stage (*p* = 0.909), or histological differentiation (*p* = 0.133).

**Figure 1:**
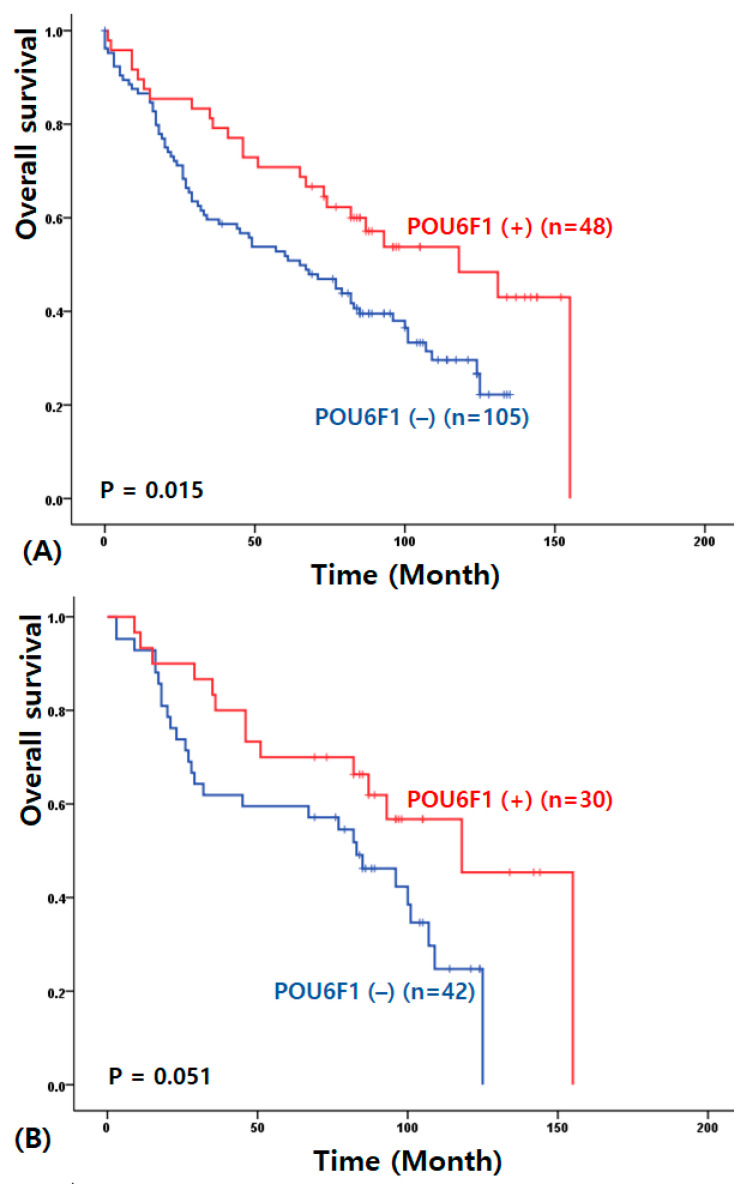

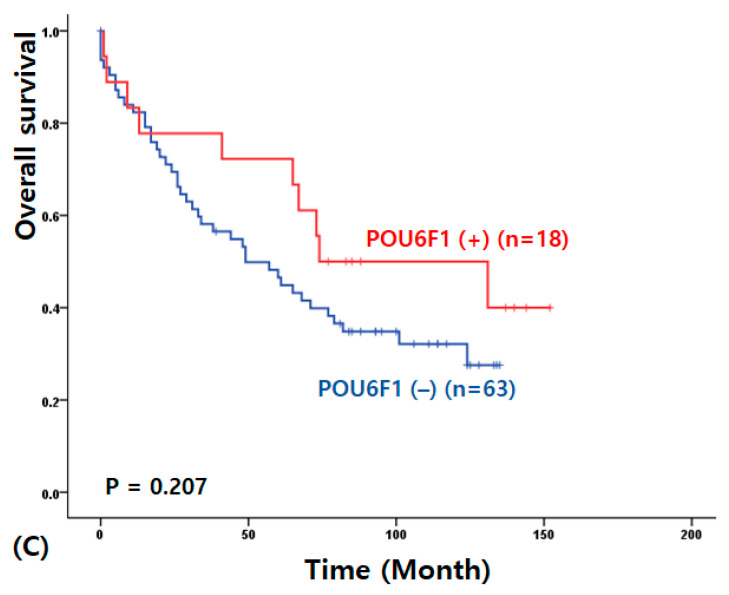
Kaplan–Meier overall survival curves according to POU6F1 expression in patients with NSCLC (**A**), lung adenocarcinoma (**B**), and lung squamous cell carcinoma (**C**).

The median follow-up period was 80.7 ± 5.1 months (range, 0–155 months). Univariate survival analysis demonstrated that overall survival (OS) in NSCLC patients was significantly associated with POU6F1 expression (100.69 ± 8.67 vs. 69.16 ± 4.99 months, *p* = 0.015; Figure 1A). When stratified by histological subtype, higher POU6F1 expression was associated with improved survival in patients with adenocarcinoma (104.67 ± 10.95 vs. 71.98 ± 7.18 months, *p* = 0.051; Figure 1B). However, POU6F1 expression was not significantly associated with survival outcomes in patients with squamous cell carcinoma (93.07 ± 13.98 vs. 66.10 ± 6.63 months, *p* = 0.207; Figure 1C). No other clinicopathological variables were found to significantly influence the prognosis of NSCLC in this analysis.

Univariate and multivariate Cox regression analyses were performed to evaluate the prognostic significance of clinicopathological variables and POU6F1 expression in patients with NSCLC. In the multivariate analysis, high POU6F1 expression was significantly associated with overall survival (HR = 0.53, *p* = 0.011). In addition, age (>60 years), advanced T stage, and lymph node metastasis were identified as independent prognostic factors (Table 2). Specifically, patients older than 60 years showed poorer survival outcomes compared with younger patients (HR = 2.15, 95% CI: 1.35–3.44, *p* = 0.001). Similarly, advanced T stage (HR = 2.75, 95% CI: 1.44–5.26, *p* = 0.002) and positive nodal status (HR = 2.88, 95% CI: 1.38–4.09, *p* = 0.002) were significantly associated with worse overall survival. These findings indicate that POU6F1 expression is an independent prognostic factor in NSCLC, along with established clinicopathological variables.

### 3.2. POU6F1 Expression in the Cancer Genome Atlas (TCGA) Data

**Figure 2:**
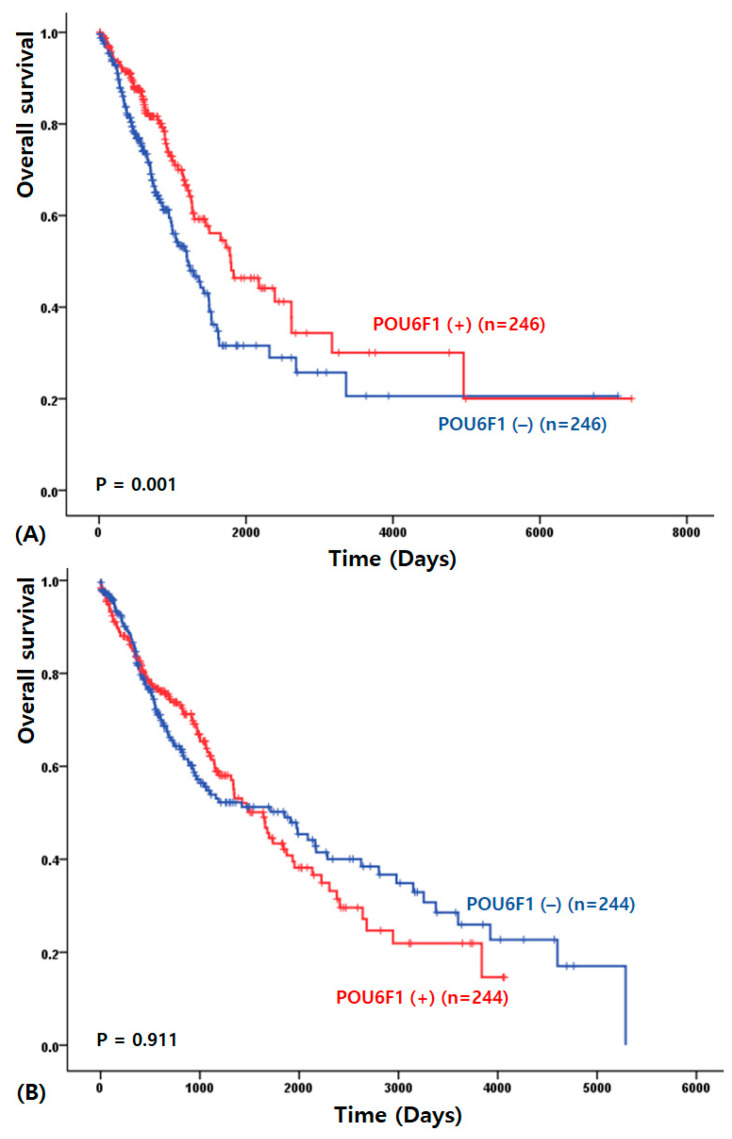
Kaplan–Meier overall survival curves according to POU6F1 expression in The Cancer Genome Atlas (TCGA) dataset of lung adenocarcinoma (**A**) and lung squamous cell carcinoma (**B**).

To validate these findings, TCGA datasets were analyzed. In the TCGA cohort, higher POU6F1 expression was significantly associated with improved survival in patients with lung adenocarcinoma (2908.81 ± 365.92 vs. 2338.17 ± 311.89 days, *p* = 0.001; Figure 2A). In contrast, survival analysis demonstrated no significant prognostic value of POU6F1 expression in squamous cell carcinoma (1839.44 ± 126.59 vs. 2229.28 ± 176.77 days, *p* = 0.911; Figure 2B).

## 4. Discussion

In the present study, we investigated the clinical and prognostic significance of POU6F1 expression in patients with non-small cell lung cancer (NSCLC). Our results demonstrated that high POU6F1 expression was significantly associated with several clinicopathological characteristics, including younger age, female sex, non-smoking status, adenocarcinoma histology, and the presence of EGFR mutations. In addition, survival analysis showed that elevated POU6F1 expression was associated with improved overall survival in patients with NSCLC. When stratified by histological subtype, this prognostic association was observed specifically in lung adenocarcinoma but not in squamous cell carcinoma, suggesting that the clinical significance of POU6F1 may differ according to the molecular characteristics of NSCLC subtypes. Furthermore, multivariate Cox regression analysis demonstrated that POU6F1 expression remained significantly associated with overall survival after adjusting for key clinicopathological factors, indicating that POU6F1 may serve as an independent prognostic factor in NSCLC.

A notable finding of this study is the consistent prognostic impact of POU6F1 expression in lung adenocarcinoma across two independent datasets. In our institutional patient cohort, higher POU6F1 expression was associated with favorable overall survival in patients with adenocarcinoma. Importantly, this observation was independently validated using the TCGA dataset, which demonstrated a similar association between elevated POU6F1 expression and improved survival outcomes in lung adenocarcinoma [12]. In contrast, no significant prognostic association was observed in squamous cell carcinoma in either dataset. The concordant results obtained from both patient tissues and the large-scale TCGA dataset strengthen the robustness and reproducibility of our findings and suggest that POU6F1 may represent a clinically meaningful prognostic biomarker specifically for lung adenocarcinoma [12]. The independent prognostic significance observed in multivariate analysis further supports the robustness of POU6F1 as a potential biomarker.

Members of the POU family of transcription factors play important roles in cellular differentiation, development, and transcriptional regulation. POU6F1, also known as BRN5, has been primarily studied in neuronal tissues, where it is involved in neural differentiation and gene regulation [9,10]. However, increasing evidence indicates that dysregulation of transcription factors can contribute to tumorigenesis by altering gene expression networks involved in cell proliferation, apoptosis, and metastasis [10,11,14,15]. Previous studies have suggested that POU6F1 may also be involved in cancer development [12,14,15]. In lung adenocarcinoma, reduced POU6F1 expression has been reported to promote tumor proliferation through the regulation of the HIF1A signaling pathway, suggesting a potential role in tumor growth and cancer-related signaling pathways.

Interestingly, POU6F1 expression in the present study was significantly associated with clinicopathological features that are typically observed in lung adenocarcinoma, including female sex, non-smoking status, and EGFR mutation status. These characteristics reflect the distinct molecular landscape of lung adenocarcinoma and have been well documented in previous studies [5,16]. POU6F1 expression may therefore be related to specific molecular subtypes of this disease. Although the precise biological mechanism remains unclear, it is possible that POU6F1 may interact with signaling pathways associated with EGFR-driven tumorigenesis or other regulatory networks involved in tumor cell proliferation and differentiation [17].

Several limitations of this study should be considered. First, this study was retrospective and conducted at a single institution. Second, the patient cohort consisted exclusively of Korean individuals, providing a relatively homogeneous genetic background. Although this may reduce variability related to ethnic heterogeneity, the molecular characteristics and clinical behavior of lung cancer may differ across populations [5,16]. Therefore, the prognostic significance of POU6F1 identified in this study may not be directly generalizable to other ethnic groups. Further studies involving larger and ethnically diverse cohorts are needed to validate the clinical significance of POU6F1 in NSCLC. In addition, functional studies were not performed to clarify the biological mechanisms underlying the association between POU6F1 expression and patient prognosis. Furthermore, our analyses were primarily based on mRNA expression levels of POU6F1, and protein-level validation was not performed. As mRNA expression does not always correlate with protein abundance, the clinical applicability of POU6F1 as a biomarker should be interpreted with caution. Future studies incorporating protein-level evaluation, such as immunohistochemical analysis, will be necessary to confirm the translational relevance of our findings.

In conclusion, our study demonstrated that POU6F1 expression is significantly associated with clinicopathological characteristics and improved survival in patients with lung adenocarcinoma. Importantly, the prognostic significance of POU6F1 was consistently observed in both our patient cohort and the independent TCGA dataset. These findings suggest that POU6F1 may serve as a potential transcript-level prognostic biomarker in NSCLC, particularly in lung adenocarcinoma. Further studies are warranted to elucidate the molecular mechanisms underlying the role of POU6F1 in lung cancer progression and to validate its clinical utility at the protein level.

## Figures and Tables

**Table 1:** Clinicopathological characteristics of POU6F1 mRNA expressions in NSCLC.

	POU6F1 Expression		
	Low	High	*p*
Total	105 (68.6)	48 (31.4)	
Age			0.027
≤60	37 (58.7)	26 (41.3)	
>60	68 (75.6)	22 (24.4)	
Sex			0.041
Female	21 (55.3)	17 (44.7)	
Male	84 (73.0)	31 (27.0)	
Type			0.021
AD	42 (58.3)	30 (41.7)	
SCC	50 (80.6)	12 (19.4)	
Others	13 (68.4)	6 (31.6)	
Smoking			0.002
(−)	17 (47.2)	19 (52.8)	
(+)	88 (75.2)	29 (24.8)	
T stage			0.351
T1	38 (76.0)	12 (24.0)	
T2	48 (68.6)	22 (31.4)	
T3	12 (60.0)	8 (40.0)	
T4	7 (53.8)	6 (46.2)	
N stage			0.449
N0	79 (69.9)	34 (30.1)	
N1	16 (59.3)	11 (40.7)	
N2	10 (76.9)	3 (23.1)	
Pathological stage			0.909
I	58 (69.9)	25 (30.1)	
II	30 (68.2)	14 (31.8)	
III	17 (65.4)	9 (34.6)	
Histology			0.133
Well/Moderate	62 (72.9)	23 (27.1)	
Poorly	20 (74.1)	7 (25.9)	
EGFR mutation			0.038
(+)	9 (45.0)	11 (55.0)	
(−)	13 (65.0)	7 (35.0)	

**Table 2:** Multivariate Cox Analysis in NSCLC.

Variable	Multivariate HR (95% CI)	*p*-Value
POU6F1 expression	0.53 (0.33–0.87)	0.011
Age	2.15 (1.35–3.44)	0.001
T stage	2.75 (1.44–5.26)	0.002
N stage	2.88 (1.38–4.09)	0.002

## Data Availability

The data presented in this study are openly available in The Cancer Genome Atlas (TCGA) at https://portal.gdc.cancer.gov/.
